# Enhanced Biosynthesis of Chlorogenic Acid and Its Derivatives in Methyl-Jasmonate-Treated *Gardenia jasminoides* Cells: A Study on Metabolic and Transcriptional Responses of Cells

**DOI:** 10.3389/fbioe.2020.604957

**Published:** 2021-01-05

**Authors:** Zebo Liu, Ali Mohsin, Zejian Wang, Xiaofeng Zhu, Yingping Zhuang, Liming Cao, Meijin Guo, Zhongping Yin

**Affiliations:** ^1^State Key Laboratory of Bioreactor Engineering, East China University of Science and Technology, Shanghai, China; ^2^Jiangxi Key Laboratory of Natural Products and Functional Foods, Jiangxi Agricultural University, Nanchang, China; ^3^Crop Breeding and Cultivation Research Institute, Shanghai Academy of Agricultural Sciences, Shanghai, China

**Keywords:** *Gardenia jasminoides* Ellis cultured cells, chlorogenic acids, methyl jasmonate, elicitation, transcriptome, transcription factors

## Abstract

Chlorogenic acid and its derivatives (CQAs) are considered as important bioactive secondary metabolites in *Gardenia jasminoides* Ellis (*G. jasminoides*). However, few studies have investigated the biosynthesis and regulation of CQAs in *G. jasminoides*. In this study, methyl jasmonate (MeJA) was used to enhance CQAs accumulation in cultured *G. jasminoides* cells. Moreover, the possible molecular mechanism of MeJA-mediated accumulation of CQAs is also explored. To this end, time-course transcriptional profiles of *G. jasminoides* cells responding to MeJA were used to investigate the mechanism from different aspects, including jasmonate (JAs) biosynthesis, signal transduction, biosynthesis of precursor, CQAs biosynthesis, transporters, and transcription factors (TFs). A total of 57,069 unigenes were assembled from the clean reads, in which 80.7% unigenes were successfully annotated. Furthermore, comparative transcriptomic results indicated that differentially expressed genes (DEGs) were mainly involved in JAs biosynthesis and signal transduction (25 DEGs), biosynthesis of precursor for CQAs (18 DEGs), CQAs biosynthesis (19 DEGs), and transporters (29 DEGs). Most of these DEGs showed continuously upregulated expressions over time, which might activate the jasmonic acid (JA) signal transduction network, boost precursor supply, and ultimately stimulate CQAs biosynthesis. Additionally, various TFs from different TF families also responded to MeJA elicitation. Interestingly, 38 DEGs from different subgroups of the MYB family might display positive or negative regulations on phenylpropanoids, especially on CQAs biosynthesis. Conclusively, our results provide insight into the possible molecular mechanism of regulation on CQAs biosynthesis, which led to a high CQAs yield in the *G. jasminoides* cells under MeJA treatment.

## Introduction

*Gardenia jasminoides* Ellis (*G. jasminoides*) is an evergreen shrub of family Rubiaceae, which is widely distributed in Southern China. Its fruits are commonly used in foods and herbal medicines in China (Debnath et al., 2011). Up to now, various bioactive components have been isolated and identified from the fruits of *G. jasminoides*, including polyphenols, iridoid glycosides, triterpenes, flavonoids, and essential oil (Zhou et al., 2010; Han et al., 2015). Among them, chlorogenic acid and its derivatives (CQAs), belonging to depsides of certain *trans*-cinnamic acids and quinic acid, are considered as important bioactive components in *G. jasminoides*. In addition, CQAs exhibit various biological activities, such as anti-inflammatory, hypolipidemic, antibiotic, and antioxidant properties, which mark them as being medically intrinsic as well (Nishi et al., 2013; Ali et al., 2017; Huang et al., 2017).

Generally, CQAs are produced by extraction from natural plants using a solvent extraction method. However, this method is not cost-effective (Fengli et al., 2009) due to the limited resources and low CQAs content in the fruits of *G. jasminoides* (He et al., 2010). In recent years, plant cell culture has been recognized as a promising alternative method for large-scale production of secondary metabolites due to its independence on the natural environment and shorter production cycle (Ochoa-Villarreal et al., 2016). In our previous studies, the callus and suspension cell culture of *G. jasminoides* were established to produce CQAs, but the low CQAs content made it not applicable for industrial-scale production (Lin et al., 2017; Liu et al., 2018). In addition, seven CQAs compounds in *G. jasminoides* cultured cell were identified via ultraperformance liquid chromatography/quadrupole time-of-flight mass spectrometry (UPLC-TOF-MS/MS), namely, 3-caffeoylquinic acid (3-CQA), 4-caffeoylquinic acid (4-CQA), 5-caffeoylquinic acid (5-CQA), 3,5-dicaffeoylquinic acid (3,5-diCQA), 4,5-dicaffeoylquinic acid (4,5-diCQA), 3,5-dicaffeoyl-4-*O*-(3-hydroxyl-3-methyl)-glutaroylquinic acid [3,5-diC(HMG)QA], and malonyl-4,5-*O*-dicaffeoylquinic acid (M-4,5-diCQA) (Liu et al., 2018). In these CQAs, 3,5-diC(HMG)QA and M-4,5-diCQA are the characteristic metabolites for *G. jasminoides* and have not been identified in other plants with high CQAs content, such as *Coffea arabica* and *Lonicera japonica*.

Jasmonic acid (JA) and methyl jasmonate (MeJA, a volatile derivative of JA) are regarded as the most important signaling molecules in plants. Above all, they not only regulate a diverse set of physiological and developmental processes but also trigger the biosynthesis of various plant secondary metabolites such as polyphenolic compounds, alkaloid, and terpenoids through extensive transcriptional reprogramming (Shoji and Hashimoto, 2011; Zhang et al., 2011; Men et al., 2013). Although we found that the exogenous MeJA significantly enhanced CQAs accumulation in cultured *G. jasminoides* cells (Liu et al., 2018), but the mechanism of MeJA-mediated CQAs biosynthesis remains so far unclear.

Due to the lack of reference genome information, it is time-consuming and laborious to investigate gene function by the traditional method in non-model organisms (Dai et al., 2011). However, RNA sequencing (RNA-seq), as a technology to obtain almost all transcriptional information of samples under certain physiological conditions, can prospectively clarify the gene function and metabolic regulation mechanism in non-model organisms due to its characteristics of “high throughput, low cost, covering a multitude of low abundance gene sequencing depth, and high sensitivity” (Todd et al., 2016). Rai et al. (2017) conducted RNA-seq on nine tissues of *L. japonica* and identified potential candidate genes that may participate in the biosynthesis of CQAs. Comparative transcriptome analysis was performed between *Eucommia ulmoides* with high and low CQAs contents, which looked into potential structural genes and transcription factors (TFs) involved in CQAs biosynthesis (Ye et al., 2019). Previously, a few studies on RNA-seq of *G. jasminoides* have been reported. Some of them focused on the physiological changes in petal senescence or melatonin treatment (Tsanakas et al., 2014; Zhao et al., 2017), and others focused on the biosynthetic pathway of carotenoids or crocins (Yang et al., 2016; Ji et al., 2017). Up to now, there have been few studies on CQAs biosynthesis in *G. jasminoides*, especially on the mechanism of MeJA-mediated CQAs biosynthesis.

In the current study, MeJA (200 μM) was utilized to increase the yield of CQAs in the cultured *G. jasminoides* cells. Additionally, the cells treated for 0 h (G0h), 8 h (G8h), 20 h (G20h), and 40 h (G40h) were selected for RNA-seq to investigate the time-course transcriptional profiles in response to MeJA elicitation. Moreover, the abundant transcriptional information was analyzed in detail to gain insight into the transcriptional changes of cells treated by MeJA. In short, this work would provide valuable information to understand the molecular mechanisms of MeJA elicitation for high CQAs production in the cultured *G. jasminoides* cells.

## Materials and Methods

### Suspension Culture of *G. jasminoides* Cells

The embryogenic *G. jasminoides* callus used in this study was obtained from Jiangxi Key Laboratory of Natural Products and Functional Food (Jiangxi Agricultural University, Nanchang, China). *G. jasminoides* cell suspension culture was established according to the method we reported previously (Liu et al., 2018). The detailed procedures were as follows: Murashige and Skoog (MS) medium was supplemented with 0.3 mg L^–1^ kinetin, 0.5 mg L^–1^ α-naphthylacetic acid, and 30 g L^–1^ sucrose (pH = 5.8) for the suspension cell culture. Six grams (fresh weight) of callus was inoculated into a 100-ml culture flask containing 40 ml MS medium, subsequently cultivated in a rotating shaker with 115 r min^–1^ at 28 ± 1°C under continuous light (illuminance = 1,000 l×), and routinely subcultured every 10 days. After 10 times of subculture, a homogeneous and rapidly growing suspension culture was obtained and used for further experiments.

### Cell Elicitation Method for High CQAs Yield

Methyl jasmonate was dissolved in 75% ethanol and sterilized by filtration prior to elicitation. *G. jasminoides* cells were cultivated in the same medium and conditions as mentioned above. On the fifth day after inoculation, the aforesaid MeJA solution was added into the culture medium at a final concentration of 200 μM, and then the cells were cultured until the scheduled harvest time (0–80 h). The harvested cells were frozen immediately in liquid nitrogen and stored at −80°C prior to use. Each treatment was carried out in triplicate.

### CQAs Determination by HPLC

The collected sample cells were dried to a constant weight. A powder sample (200 mg) was extracted with 10 ml of methanol/water (7:3, v/v) in an ultrasound extractor for 50 min. The extracts were determined by high-performance liquid chromatography (HPLC) according to our previously reported method (Liu et al., 2018). Seven CQAs, namely, 3-CQA, 4-CQA, 5-CQA, 3,5-diCQA, 4,5-diCQA, 3,5-diC(HMG)QA, and M-4,5-diCQA, were quantified.

### RNA Extraction, Library Construction, and Illumina Sequencing (RNA-seq)

Four samples, in which cells were treated by MeJA for 0, 8, 20, and 40 h, were prepared by mixing three replicate samples of the harvested cells before RNA extraction. Total RNA was extracted using a Total RNA Extractor (SanGon, Shanghai, China) according to the manufacturer’s instructions. mRNA was enriched by Oligo(dT) and used to construct a non-stranded RNA-seq transcriptome library. After fragmentation, reverse transcription, 3′ add A, adaptor ligation, and PCR amplification, the cDNA library was obtained and then subjected to the Illumina HiSeq X Ten (PE150) platform with an average insert length of 350 bp for paired-end sequencing with one technical replicate. In addition, total cDNA libraries of cell samples exposed to MeJA for 0, 8, 20, and 40 h were designated as “G0h,” “G8h,” “G20h,” and “G40h”, respectively, in the current study.

### *De novo* Assembly and Gene Functional Annotation

To obtain the clean reads, raw reads were filtered by removing adapter sequences, low-quality reads (quality value < 20), and the reads with a length less than 35 bp. Prior to assembly, each library mentioned in section “RNA Extraction, Library Construction, and Illumina Sequencing (RNA-seq)” was pooled. Then, *de novo* assembly was conducted by software Trinity (version number: 2.4.0) with a min_kmer_cov parameter of 2. High-quality reads were subjected to module Chrysalis to obtain unique contigs, which were clustered to generate Bruijn. Module Butterfly was used to process Bruijn to obtain full-length transcripts with alternative splicing and to distinguish paralogs. After transcript de-redundancy, the longest ones of each transcript cluster were regarded as isoforms representing unigenes (Grabherr et al., 2011).

All assembled unigenes were used as query sequences to search against publicly available protein databases, including National Center for Biotechnology Information (NCBI) non-redundant protein sequences (NR) and Swiss-Prot, for homology comparison using NCBI Blast+ program (version number: 2.28) with identity set at >30% and a cutoff *E*-value of 10^–5^ (Conesa et al., 2005). By using WEGO software, the assembled unigenes were annotated on Gene Ontology (GO) and Clusters of Orthologous Groups of proteins (COG/KOG) for gene function prediction and classification (Ye et al., 2006). The KEGG Automatic Annotation Server (KAAS, version number: 2.1) was used to assign unigenes based on the Kyoto Encyclopedia of Genes and Genomes (KEGG) database for the secondary metabolic pathway annotation (Kanehisa et al., 2007). Annotation of TFs and transcriptional regulators was conducted by blasting the assembling unigenes against PlnTFDB V3.0^1^ with *E*-values < 1e−5. When a conflict occurred among the database results, the priority order of alignments was Nr, Swiss-Port, KEGG, GO, and then COG.

### Analysis of Differentially Expressed Genes

To normalize the gene expression levels, the fragment per kilobase of exon model per million mapped reads (FPKM) method was adopted on the RSEM software (version number: 1.0) (Mortazavi et al., 2008). The differential expression analysis of any two groups was analyzed using the previously reported method (Audic and Claverie, 1997) with Benjamini and Hochberg false discovery rate (FDR). Log_2_[fold change (FC)] > 1 (FDR < 0.001) was set as the threshold to identify upregulated differentially expressed genes (DEGs), and Log_2_(FC) < −1 (FDR < 0.001) was set as the threshold to identify downregulated DEGs (Reiner et al., 2003). To further analyze DEGs involved in secondary metabolism, KEGG significant enrichment was performed by comparing DEGs to the entire background of all KEGG-associated unigenes with a hypergeometric test (*p* < 0.05, the *p*-value of the hypergeometric test represents the pathway significance; in other words, the lower the *p*-value, the more significant the pathway changes), and then significantly overrepresented metabolic and signal transduction pathways were identified and ranked (Rai and Saito, 2016). Comparative analyses between G0h and G8h, G20h, or G40h were designated as “G0h vs G8h,” “G0h vs G20h,” and “G0h vs G40h,” respectively, in the current study.

### Validation of DEG Expression Level by qRT-PCR

Total RNA was isolated from *G. jasminoides* cells elicited by MeJA for different periods (0, 8, 20, and 40 h) using TRIzol^®^ reagent (Invitrogen) following the manufacturer’s recommendations. After treatment with DNase I (SanGon, China), 1 μg of RNA was used in reverse transcription with Revert Aid Premium Reverse Transcriptase kits (Thermo Scientific^TM^, China). qRT-PCR was performed using SG Fast qPCR Master Mix (ABI, United States) and ran on Step One Plus Real-Time PCR Detection System (ABI, United States). The cycling conditions were initial denaturation at 95°C for 3 min, followed by 45 cycles of 95°C for 3 s, 60°C for 30 s, and 72°C for 15 s. Fifteen selected unigenes were validated by qRT-PCR experiments, and qRT-PCR reactions were carried out in triplicate. Gene-specific primer pairs (Supplementary Table 1) were designed by Primer 6.0 software, and a polyubiquitin (UBQ5) gene (c15827_g1) served as the reference gene because it was relatively stable across all time points. Gene relative expression was calculated by the 2^−ΔΔCT^ method (Livak and Schmittgen, 2001), and all data were expressed as means ± SD after normalization. To evaluate the quality of RNA-seq data, correlation analyses of gene relative expression between qRT-PCR and RNA-seq were performed with Pearson correlation coefficient (two-tailed test, confidence interval = 95%).

## Results

### CQAs Accumulation in MeJA-Treated *G. jasminoides* Cells

**FIGURE 1:**
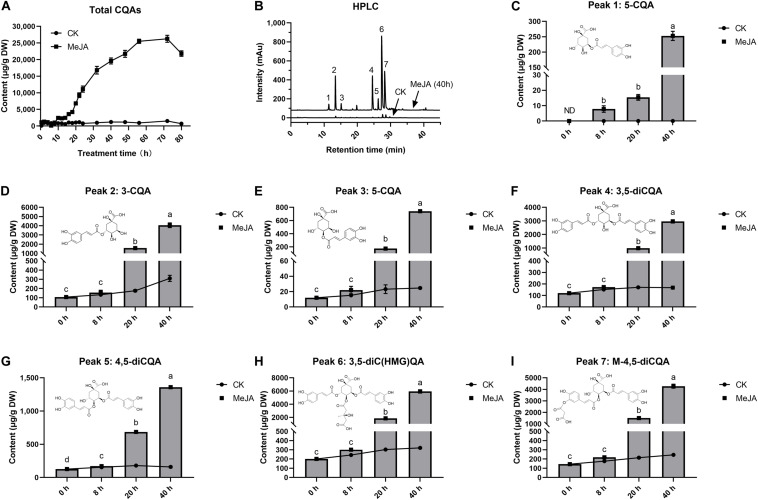
Effects of MeJA (200 μM) on CQAs accumulation in cultured *G. jasminoides* cells. **(A)** CQAs content change profile. **(B)** HPLC of the extracts from the control cells and MeJA-treated cells (*Peak 1*: 5-CQA; *Peak 2*: 3-CQA; *Peak 3*: 4-CQA; *Peak 4*: 3,5-diCQA; *Peak 5*: 4,5-diCQA; *Peak 6*: 3,5-diC(HMG)QA; *Peak 7*: M-4,5-diCQA.); **(C–I)** content change profile for individual CQAs from Peaks 1 to 7. Different superscript lowercase letters “a–d” indicates a significant difference between data at *p* < 0.05. Statistical analysis was performed with ANOVA followed by Duncan’s multiple range test (DMRT).

The effect of MeJA on intracellular accumulation of CQAs was examined at 80 h after elicitation (Figure 1A). After 8 h, MeJA began to stimulate a rapid increase in CQAs. These CQAs remained in relatively high concentrations until the 72nd hour of elicitation, the maximum of which was 21.7 times (26.26 mg g^–1^) more than that of control, whereas total CQAs content varied in a little range (0.71–1.21 mg g^–1^) without MeJA elicitation (CK). The selected cell samples were exposed to MeJA for 0, 8, 20, and 40 h, and both total CQAs content and its fold changes were highest in G40h (19.59 mg g^–1^, 17.65 times), followed by G20h (6.81 mg g^–1^, 6.49 times), G8h (1.05 mg g^–1^, 1.57 times), and G0h (0.711 mg g^–1^, 1 time). Based on the results, it was observed that MeJA made a differential contribution to individual CQAs production (Figures 1B–I). MeJA stimulated abundant accumulation of 3-CQA, 3,5-diCQA, 3,5-diC(HMG)QA, and M-4,5-diCQA, while a relatively slight increase occurred in the accumulation of 4-CQA, 5-CQA, and 4,5-diCQA. Taking G40h for example, the total content of 3-CQA, 3,5-diCQA, 3,5-diC(HMG)QA, and M-4,5-diCQA could reach up to 17.23 mg g^–1^, which accounted for 87.95% of total CQAs content. Thus, the above results indicated that MeJA treatment led to differential metabolic characteristics and significant accumulation of CQAs in *G. jasminoides* cells.

### RNA-seq and *de novo* Assembly

**FIGURE 2:**
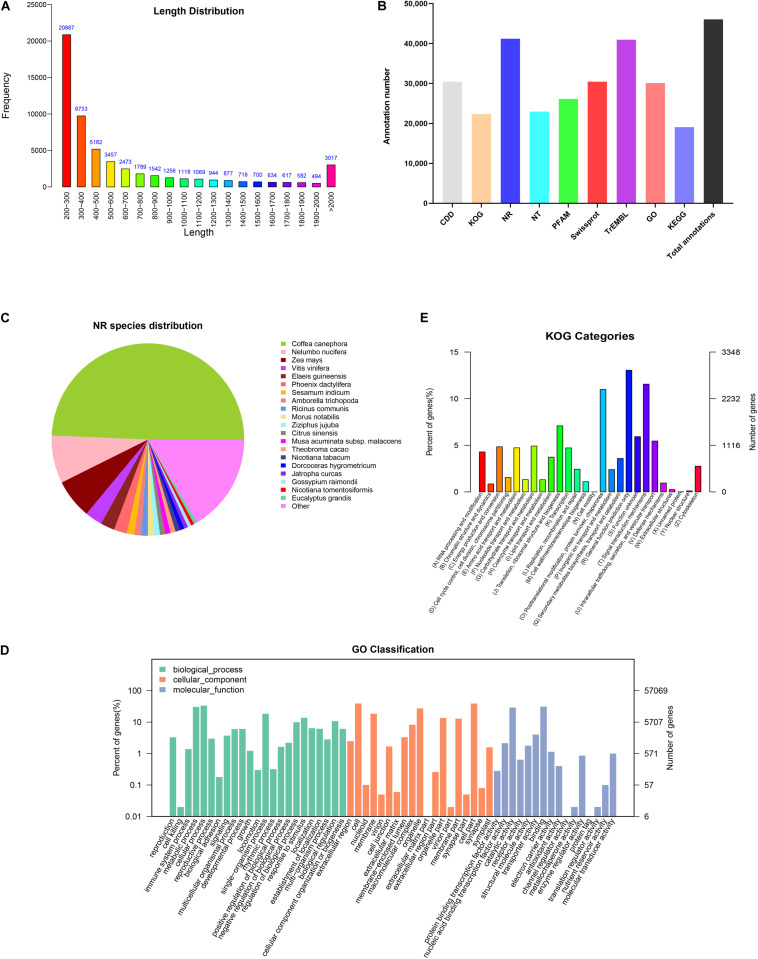
Functional annotations of unigenes from cultured *G. jasminoides* cells. **(A)** The length distribution of assembled unigenes; **(B)** unigene annotation in the public databases; **(C)** NR classification; **(D)** GO classification; **(E)** KOG classification.

In order to comprehensively elucidate the time-course transcriptome of *G. jasminoides* cells under MeJA elicitation, four cDNA libraries from the cells stimulated by MeJA for 0, 8, 20, and 40 h were sequenced via the Illumina HiSeq × Ten platform, which generated 36.94, 40.39, 40.92, and 38.96 million raw reads, respectively (Table 1). After the quality control of raw reads, the clean reads were obtained with GC percentage ranging from 51.44 to 55.22% (Table 1). Subsequently, the Trinity program was used to assemble all clean reads into 63,724 transcripts with an N50 of 1,153 bp and an average length of 703 bp, which were then joined into 57,069 unigenes with an N50 of 1,051 bp and an average length of 655 bp (Table 2). As shown in Figure 2A, all unigenes were longer than 200, and 200–300 bp was the most prominent length distribution interval. Among all unigenes, there were 35,782 (62.70%) unigenes with lengths shorter than 500 bp, 10,517 (18.50%) unigenes with lengths ranging from 500 to 1,000 bp, and 10,770 (18.88%) unigenes with lengths more than 1,000 bp. Overall, the quality of assembly results was high enough to conduct further analyses.

**TABLE 1:** Summary of transcriptome sequencing of cultured *G. jasminoides* cells.

Sample	G0h	G8h	G20h	G40h
Total raw reads	36,935,766	40,388,174	40,920,732	38,961,636
Total clean reads	33,408,764	36,779,394	37,295,714	35,578,996
Q20 base ratio (%)	97.62	97.67	97.73	97.70
Q30 base ratio (%)	91.87	92.01	92.17	92.12
Error (%)	0.00	0.00	0.00	0.00
GC base ratio (%)	54.42	55.22	54.22	51.44

*Q20, the proportion of nucleotides with a quality value larger than 20 in reads; Q30, the proportion of nucleotides with a quality value larger than 30 in reads (the cell samples exposed to MeJA for 0, 8, 20, and 40 h were designated as “G0h,” “G8h,” “G20h,” and “G40h,” respectively).*

**TABLE 2:** Summary of the sequence assembly after Illumina sequencing.

	Transcript	Unigene
All number	637,24	57,069
200–500 bp	37,496	35,782
500–1,000 bp	12,482	10,517
≥1,000 bp	13,746	10,770
Max length	13,000	13,000
Min length	201	201
All length	44,803,051	37,411,700
Mean length	703.08	655.55
N50 (bp)	1,153	1,051

### Functional Annotation and Classification of Unigenes

In order to investigate the functions of assembled unigenes, the entire unigenes were searched against the NR, Pfam, Swiss-Prot, GO, KOG, and KEGG databases. The annotation ratios of each database are shown in Figure 2B. A total of 46,054 unigenes (80.7%) were aligned to homologous sequences in those public databases.

A total of 41,201 unigenes were annotated in NR databases (Figure 2B) and compared to known nucleotide sequences from other plant species for specie homology analysis (Figure 2C). A dominant homology existed between the assembled unigenes from *G. jasminoides* and known nucleotide sequences from *Coffea canephora* (49.3%), a plant in Rubiaceae family, followed by *Nelumbo nucifera* (8.0%), *Zea mays* (6.6%), and *Vitis vinifera* (2.9%).

Gene Ontology is an international classification system for standardized gene functions, aiming to comprehensively describe the properties of genes and their products in any organism. Based on the NR annotation, 30,119 unigenes were classified into three major categories such as “Biological process,” “Cellular component,” and “Molecular function,” which were further grouped into 58 sub-categories (Figure 2D). Among those categorized in the “Biological process,” numerous unigenes were associated with “cellular process” (19,017 unigenes, 63.14%), “metabolic process” (17,347 unigenes, 57.59%), and “single-organism process” (10,508 unigenes, 34.89%). Under the “Cellular component” category, the three top-hit sub-categories were “cell” (22,218 unigenes, 73.77%), “cell part” (22,216 unigenes, 73.76%), and “organelle” (15,524 unigenes, 51.54%). Within the “Molecular function” category, unigenes were mostly enriched in “binding” (17,660 unigenes, 58.63%), “catalytic activity” (16,564 unigenes, 55.00%), and “transporter activity” (2,291 unigenes, 7.61%).

The KOG database offers an annotation system of orthologous relationship for eukaryotic complete genomes, in which every protein is assumed to be evolved from an ancestor protein. To further elucidate and classify the *G*. *jasminoides* transcriptome, 22,319 unigenes were distributed into 26 functional groups based on the KOG database (Figure 2E). Among these unigenes, 2,913 (13.05%) unigenes were dominantly enriched in the “General function prediction only” category, followed by “Signal transduction mechanisms” (2,579 unigenes, 11.56%) and “Posttranslational modification, protein turnover, chaperones” (2,456 unigenes, 11.00%). Only a few unigenes were grouped into “Unnamed protein” (three unigenes, 0.01%) and “Cell motility” (one unigene, 0.003%). However, there were still 1,323 (5.93%) unigenes categorized to “Function unknown.”

### Identification of DEGs During the MeJA-Treated Process

**FIGURE 3:**
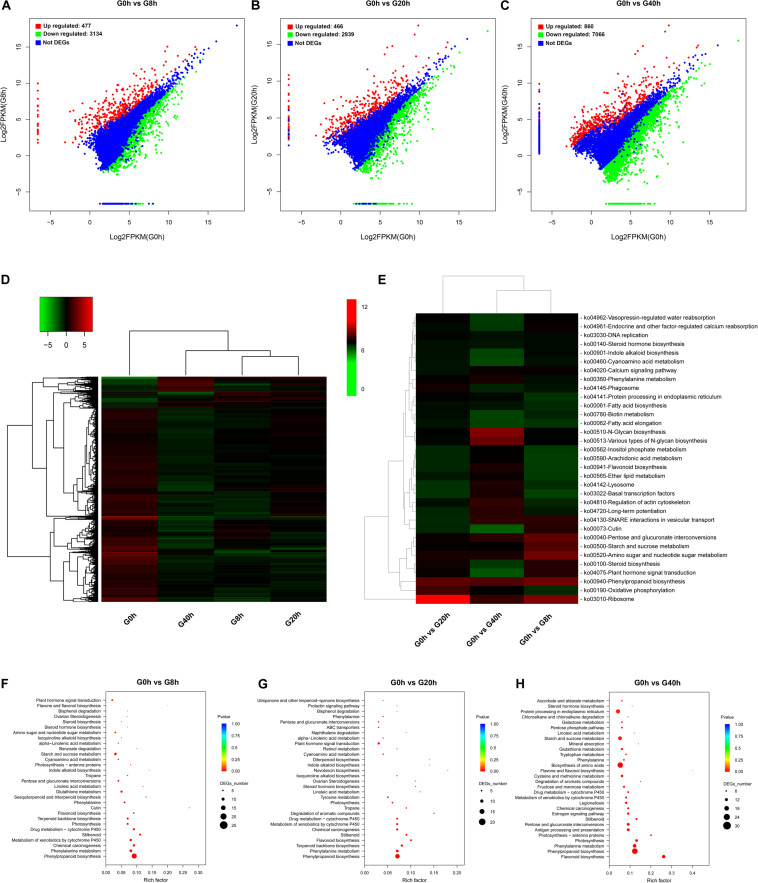
DEG identification and their KEGG enrichment analysis: DEG scatter plots of **(A)** “G0h vs G8h,” **(B)** “G0h vs G20h,” and **(C)** “G0h vs G40h.” **(D)** Heatmap of DEG expression profile in all samples [the expression values of DEGs were normalized by log_2_(FPKM + 1)]; **(E)** KEGG enrichment heatmap of all DEGs [the value of each rectangle was normalized by –log(*p*-value), and a higher value represented more significant enrichment]; KEGG enrichment on upregulated DEGs in **(F)** “G0h vs G8h,” **(G)** “G0h vs G20h,” and **(H)** “G0h vs G40h” (the rich factor indicated the ratio of DEGs to all annotated genes in one pathway, and the lower *p*-value represented more significant enrichment).

The pathway enrichment analysis was implemented by searching for DEGs against the KEGG database to get insight into mechanisms of biological functions and detect the genes responsible for cross-talk between jasmonate (JAs) signaling and key biochemical pathways. To identify DEGs and analyze their time-course expression profile during the MeJA-treated process, we analyzed the following comparisons: G0h vs G8h, G0h vs G20h, and G0h vs G40h, in which 3,611 (477 upregulated/3,134 downregulated), 3,405 (466 upregulated/2,939 downregulated), and 7,926 (860 upregulated/7,066 downregulated) DEGs were identified, respectively (Figures 3A–C).

The expression profiles based on the log_2_(fold change) values revealed a total of 8,264 DEGs in G0h vs G8h, G0h vs G20h, and G0h vs G40h (Figure 3D). Furthermore, KEGG enrichment and cluster analyses on all DEGs were conducted to investigate the major biochemical metabolism and signal transduction pathways in which DEGs participated (Rai et al., 2016). All DEGs were individually enriched into 296 KEGG pathways, and the top 33 most significantly enriched pathways were displayed in Figure 3E. KEGG enrichment results showed that the top five most significantly enriched pathways were “Ribosome,” “Oxidative phosphorylation,” “Phenylpropanoid biosynthesis,” “Plant hormone signal transduction,” and “Steroid biosynthesis.” Besides, other pathways involved in primary metabolism or secondary metabolism, e.g., “Fatty acid biosynthesis,” “Steroid hormone biosynthesis,” “Flavonoid biosynthesis,” and “Indole alkaloid biosynthesis,” were significantly enriched as well.

Generally, accumulation of the secondary metabolites was enhanced as a result of the increasing metabolic flux. Thus, the upregulated DEGs were subjected to the KEGG database for significant enrichment to further clarify the molecular mechanism of MeJA elicitation on the secondary metabolism (Figures 3F–H). Pathway enrichment on upregulated DEGs was significantly assigned to “Phenylpropanoid biosynthesis” and “Phenylalanine metabolism,” which were closely bound up with CQAs biosynthesis, in all comparisons. Other key biosynthesis pathways, including “Flavonoid biosynthesis,” “Terpenoid backbone biosynthesis,” “Sesquiterpenoid and triterpenoid biosynthesis,” “Indole alkaloid biosynthesis,” and “Isoquinoline alkaloid biosynthesis,” which provide precursors for CQAs biosynthesis or other therapeutic secondary metabolites, were significantly enriched as well. Moreover, the pathways in response to MeJA signaling (e.g., “alpha-Linolenic acid metabolism” and “Plant hormone signal transduction”) were also shown to be highly enriched. Consequently, we make further efforts to focus on JAs biosynthesis and its signal transduction pathway, key precursor biosynthesis pathway, phenylpropanoid biosynthesis pathway, transporters, and TFs.

### Analysis of DEGs Involved in JAs Biosynthesis and Signal Transduction Pathway

**FIGURE 4:**
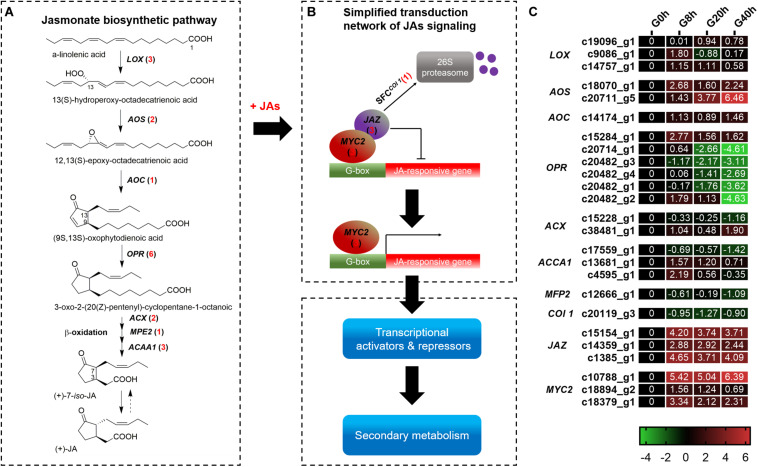
The JAs biosynthetic pathway and JAs signal transduction network. **(A)** JAs biosynthetic pathway; **(B)** transduction network of JAs signaling; **(C)** expression heatmap of DEGs in each sample. Numbers in parentheses following each gene name indicate the number of corresponding DEGs. The expression values of DEGs were normalized by Log2 of (value under treatment +1)/(value at 0hr + 1) for each time point. (*LOX*, *lipoxygenase*; *AOS*, *hydroperoxide dehydratase*; *AOC*, *allene oxide cyclase*; *OPR*, *12-oxophytodienoic acid reductase*; *ACX*, *acyl-CoA oxidase*; *MPE2*, *enoyl-CoA hydratase/3-hydroxyacyl-CoA dehydrogenase*; *ACAA1*, *acetyl-CoA acyltransferase 1*.)

A total of 25 DEGs (Figure 4) were identified, in which 18 and 7 DEGs were subjected to JAs biosynthesis and signal transduction pathways, respectively. In the JAs biosynthesis pathway (Figure 4A), most of the DEGs in these gene families were more highly expressed in G8h, G20h, and G40h than in G0h. Among these DEGs, 12 DEGs, namely, *lipoxygenase* (*LOX*, three DEGs), *allene oxide synthase* (*AOS*, two DEGs), *allene oxide cyclase* (*AOC*, one DEG), *12-oxophytodienoic acid reductase* (*OPR*, three DEGs), *acyl-CoA oxidase* (*ACX*, one DEG), and *acetyl-CoA acyltransferase 1* (*ACCA1*, two DEGs), were all continuously upregulated, but five DEGs corresponding to *OPR* (three DEGs), *ACCA1* (one DEG), and *enoyl-CoA hydratase/3-hydroxyacyl-CoA dehydrogenase* (*MEP2*, one DEG) were slightly downregulated in G8h, G20h, and G40h. Moreover, the ascending order of transcript abundance for upregulated DEGs was generally as follows: G0h, G40h, G20h, and G8h (Figure 4C). These results suggested that the extensive changes in transcription levels for these 18 DEGs involved in JAs biosynthesis might influence endogenous JAs accumulation.

In terms of DEGs in the plant hormone signal transduction pathway (Supplementary Figure 1), a total of 87 DEGs were identified. Overall, the DEGs in the signal transduction [auxins (AUX), cytokinins (CK), brassinosteroids (BR), gibberellins (GA), abscisic acid (ABA), ethylene (ET), and salicylic acid (SA)] except for JA signaling showed a downward trend in G8h, G20h, and G40h compared to that in G0h (Supplementary Figure 2). As for JA signaling, six DEGs comprising *MYC2* (three DEGs) and *JAZ* (three DEGs) showed a significant increase of transcript abundance from G0h and remained in extremely high expressions in G8h, G20h, and G40h. For instance, a *MYC2* DEG (c10788_g1) and a *JAZ* DEG (c1385_g1) were most highly expressed (Figure 4C), in which the gene expression (FPKM value) in G40h could be 105.8 and 22.41 times those in G0h, respectively. Moreover, one DEG encoding *Col 1* protein displayed a downward trend in G8h, G20h, and G40h compared to that in G0h. The results revealed that the exogenous MeJA treatment could modulate the expression of genes for endogenous JA biosynthesis and trigger the JAs signal transduction network.

### Analysis of DEGs Engaged in Biosynthesis of Precursors for Phenylpropanoids

**FIGURE 5:**
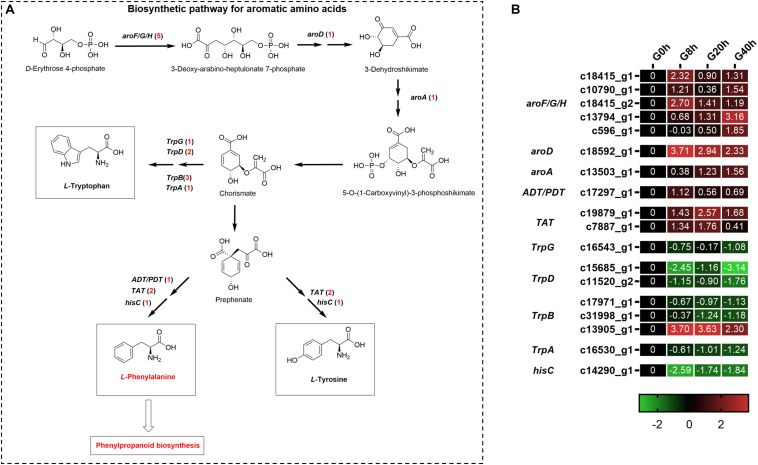
Putative biosynthesis pathways **(A)** and expression heatmap of DEGs **(B)** for aromatic amino acids (***L***-phenylalanine, ***L***-tyrosine, and ***L***-tryptophan); numbers in parentheses following each gene name indicate the number of corresponding DEGs. Some structural genes that were not DEGs were not shown in **A**. The expression values of DEGs were normalized by Log2 of (value under treatment +1)/(value at 0hr + 1) for each time point. (*aroF/G/H*, *3-deoxy-7-phosphoheptulonate synthase*; *aroD*, *3-dehydroquinate dehydratase I*; *aroA*, *3-phosphoshikimate 1-carboxyvinyltransferase*; *ADT/PDT*, *arogenate/prephenate dehydratase*; *TAT*, *tyrosine aminotransferase*; *TrpG*, *anthranilate synthase component II*; *TrpD*, *anthranilate phosphoribosyltransferase*; *TrpB*, *tryptophan synthase beta chain*; *TrpA*, *tryptophan synthase alpha chain*; *hisC*, *histidinol-phosphate aminotransferase*.)

Aromatic amino acids, including phenylalanine, tyrosine, and tryptophan, were generated via the shikimate pathway in plants and microbes (Figure 5A), and phenylalanine was a vital precursor for phenylpropanoid biosynthesis. Our transcriptome data showed that 18 DEGs were annotated to the biosynthetic pathway for aromatic amino acids under MeJA treatment (Figure 5B). Among them, there were 10 DEGs found to be upregulated and involved in phenylalanine and tyrosine biosynthesis, namely, *3-deoxy-7-phosphoheptulonate synthase* (*aroF/G/H*, five DEGs), *3-dehydroquinate dehydratase I* (*aroD*, one DEG), *3-phosphoshikimate 1-carboxyvinyltransferase* (*aroA*, one DEG), *arogenate/prephenate dehydratase* (*ADT/PDT*, one DEG), and *tyrosine aminotransferase* (*TAT*, two DEGs). Another eight DEGs showed downregulated expressions, in which seven DEGs participated in tryptophan biosynthesis, namely, *anthranilate synthase component II* (*TrpG*, one DEG), *anthranilate phosphoribosyltransferase* (*TrpD*, two DEGs), *tryptophan synthase beta chain* (*TrpB*, three DEGs), and *tryptophan synthase alpha chain* (*TrpA*, one DEG). The above-mentioned results indicated that MeJA could stimulate the expression of genes for the phenylalanine and tyrosine biosynthesis and, meanwhile, downregulated the expression of genes in the branching pathway (tryptophan biosynthesis), which enhanced the supply of precursors for phenylpropanoid biosynthesis.

### Analysis of DEGs Related to Phenylpropanoid Biosynthesis and Transporter

**FIGURE 6:**
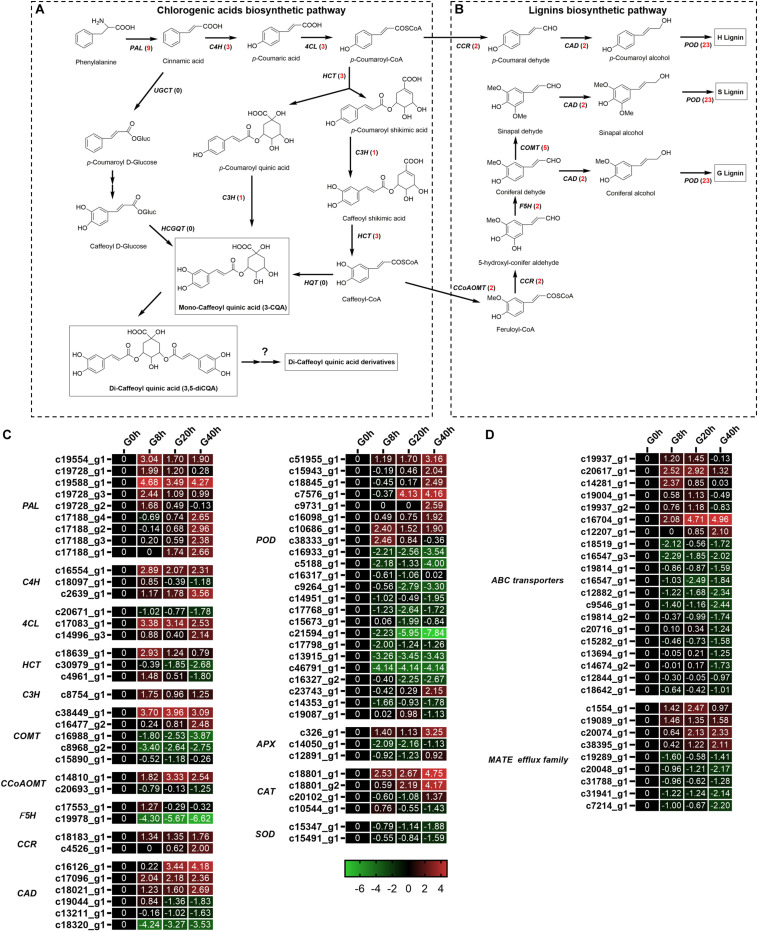
Putative biosynthesis pathways for CQAs **(A)** and lignins **(B)**. Expression heatmap of DEGs in CQAs and lignin biosynthesis **(C)** and transporters **(D)**. Numbers in parentheses following each gene name indicate the number of corresponding DEGs. The expression values of DEGs were normalized by Log2 of (value under treatment +1)/(value at 0hr + 1) for each time point. (*PAL*, *phenylalanine ammonia lyase*; *C4H*, *cinnamate 4-hydroxylase*; *4CL*, *4-coumarate CoA ligase*; *HCT*, *hydroxycinnamoyl CoA shikimate/quinate hydroxycinnamoyl transferase*; *C3H*, *p-coumarate 3′-hydroxylase*; *HQT*, *hydroxycinnamoyl CoA quinate hydroxycinnamoyl transferase*; *UGCT*, *UDP glucose:cinnamate glucosyl transferase*; *HCGQT*, *hydroxycinnamoyl D-glucose:quinate hydroxycinnamoyl transferase*; *COMT*, *caffeic acid 3-*O*-methyltransferase*; *CCoAMT*, *caffeoyl-CoA* O*-methyltransferase*; *F5H*, *ferulate-5-hydroxylase*; *CCR*, *cinnamoyl-CoA reductase*; *CAD*, *cinnamyl-alcohol dehydrogenase*.)

Chlorogenic acids derived from phenylpropanoid biosynthesis pathway are a class of depsides of certain *trans*-cinnamic acids and quinic acids (Figure 6A). A total of 19 DEGs (Figures 6A–C) were identified to be involved in CQAs biosynthesis, and 17 of these DEGs were found to be significantly upregulated in G8h, G20h, and G40h compared to those in G0h, namely, *phenylalanine ammonia-lyase* (*PAL*, nine DEGs), *trans-cinnamate 4-monooxygenase* (*C4H*, three DEGs), *4-coumarate-CoA ligase* (*4CL*, two DEGs), *shikimate O-hydroxycinnamoyltransferase* (*HCT*, two DEGs), and *coumaroyl quinate/coumaroyl shikimate 3′-monooxygenase* (*C3H*, one DEG). In particular, the expression of the *PAL* family showed the most significant upregulation, and *PAL* (c19588_g1) was upregulated over 28.89-fold in G8h compared with that in G0h. However, none of the *hydroxycinnamoyl CoA quinate hydroxycinnamoyl transferase (HQT)* gene was even annotated by the BLAST program in our transcriptome data. These results implicated a correlation between the increasing metabolic flux and CQAs accumulation owing to MeJA elicitation.

Interestingly, a total of 40 DEGs (Figure 6C) related to lignin biosynthesis [e.g., *caffeic acid 3-*O*-methyltransferase* (*COMT*), *caffeoyl-CoA* O*-methyltransferase* (*CCoAMT*), *ferulate-5-hydroxylase* (*F5H*), *cinnamoyl-CoA reductase* (*CCR*), *cinnamyl-alcohol dehydrogenase* (*CAD*), and *peroxidase* (*POD*)] were identified, and 18 of these DEGs were upregulated in G8h, G20h, and G40h compared with that in G0h. Our result was in good agreement with the study of Chen et al. (2015), which indicated that MeJA treatment led to increased expression of genes involved in lignan biosynthesis (*IiCAD* and *IiCCR*) and accumulation of several lignans in *Isatis indigotica* hairy roots.

In plants, the transporter system and subcellular localization are major elements in the process of biosynthesis regulation, transport, and accumulation of secondary metabolites (de Brito Francisco and Martinoia, 2018). The *ATP-binding cassette* (*ABC*) transporter and *multidrug and toxin extrusion* (*MATE*) protein were reported to be the main transporters for phenylpropanoid. In our transcriptome data (Figure 6D), 20 DEGs were subjected to *ABC* transporters, while nine DEGs were classified into *MATE*. Among these DEGs, four *ABC* transporters and four *MATEs* were upregulated in “G0h vs G8h,” “G0h vs G20h,” and “G0h vs G40h,” illustrating that MeJA elicitation could show a significant influence on the expression of transporter genes.

### Identification and Classification of DEGs Associated With TFs

Due to powerful regulation, TFs play a pivotal role in plant reproduction, bioactive metabolite synthesis, and response to environmental stress and thus have become a research hotspot recently (Sun et al., 2013). Further identification of TFs will help us gain a better understanding of gene regulatory networks, especially for CQAs biosynthesis in MeJA-treated *G. jasminoides* cells.

**FIGURE 7:**
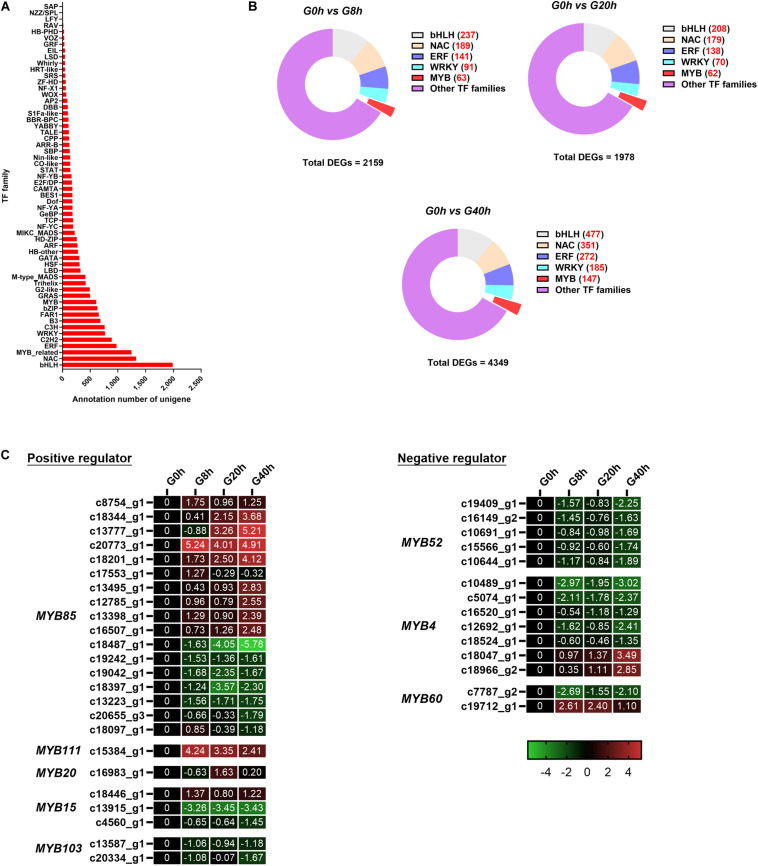
**(A)** TF annotation for all unigenes; **(B)** DEGs of the TF family in “G0h vs G8h,” “G0h vs G20h,” and “G0h vs G40h”; **(C)** expression heatmap of DEGs in different subgroups from MYB family. The expression values of DEGs were normalized by Log2 of (value under treatment +1)/(value at 0hr + 1) for each time point.

In this work, a total of 17,807 unigenes were annotated in the Plant Transcription Factor Database (PlnTFDB) and classified to 55 known TF families (Figure 7A). The most representative TF family was the basic helix–loop–helix (bHLH) family (1,988 unigenes), followed by NAC (1,326 unigenes), MYB-related (1,244 unigenes), ERF (975 unigenes), and C2H2 families (888 unigenes).

Some TF families (e.g., MYB, bHLH, NAC, ERF, and WRKY families), especially MYB, usually participate in the regulation on the secondary metabolism. Thus, DEGs in the above-mentioned TF families were classified for further analysis (Figure 7B). Among these DEGs, there were 63 (9 up/54 down), 62 (12 up/50 down), and 147 (25 up/122 down) DEGs in the MYB family of “G0h vs G8h,” “G0h vs G20h,” and “G0h vs G40h,” respectively. Furthermore, a total of 38 DEGs, which could play positive/negative regulation on CQAs biosynthesis, were identified and listed in Figure 7C. In the positive regulators, the *MYB85* family contained the maximum number of DEGs (17 unigenes), and nine of those DEGs displayed an upregulated expression in G8h, G20h, and G40h compared with those in G0h. Besides, *MYB111* (one DEG), *MYB20* (two DEGs), and *MYB15* (one DEG) were found to be upregulated as well. In terms of the negative regulators (Figure 7C), 14 DEGs were identified, in which 11 downregulated DEGs were classified into *MYB60* (one DEG), *MYB52* (five DEGs), and *MYB4* (five DEGs) families. To this end, these results revealed that MeJA could significantly modulate the expression of *MYB* TFs, which might participate in phenylpropanoid biosynthetic regulation.

### Validation of DEG Expression Level by qRT-PCR

**FIGURE 8:**
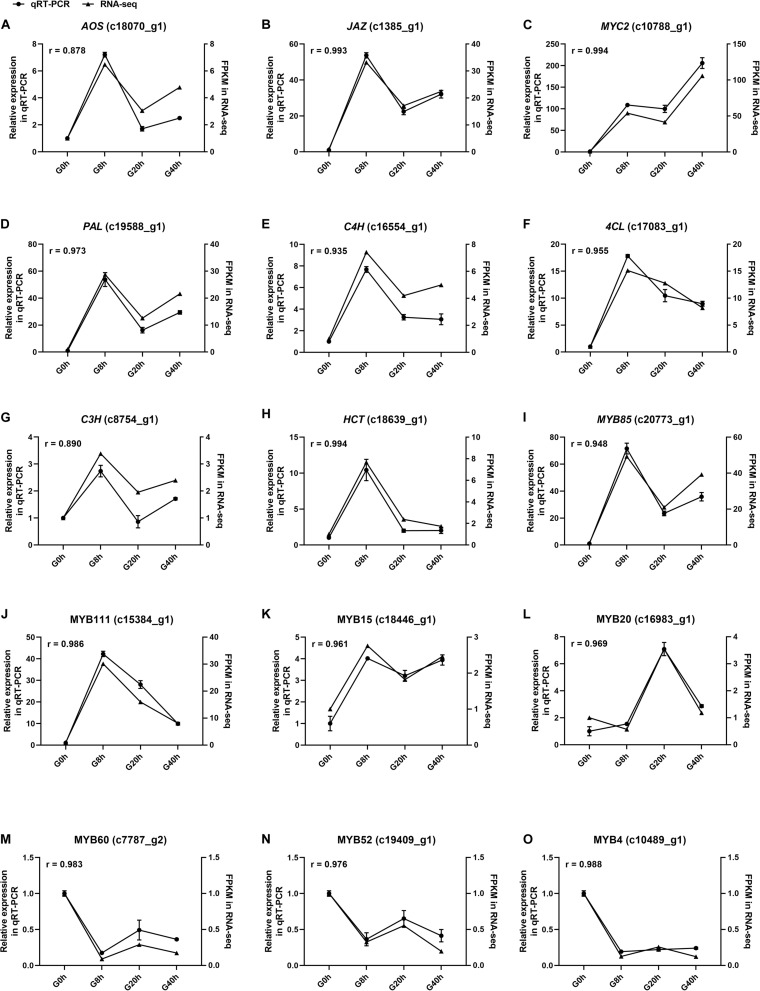
The expression level of fifteen selected genes **(A–O)** in cultured *G. jasminoides* cells under MeJA elicitation by qRT-PCR [the cell samples exposed to 200 μM MeJA (final concentration) for 0, 8, 20, and 40 h were designated as “G0h,” “G8h,” “G20h,” and “G40h,” respectively. “*r*” meant Pearson correlation coefficient].

Fifteen DEGs involved in JA biosynthesis and signal transduction [*AOS* (c18070_g1), *JAZ* (c1385_g1), *MYC2* and (c10788_g1)], CQAs biosynthesis [*PAL* (c19588_g1), *C4H* (c16554_g1), *4CL* (c17083_g1), *C3H* (c8754_g1), and *HCT* (c18639_g1)], and TF that modulates the CQAs biosynthesis [*MYB85* (c20773_g1), *MYB111* (c15384_g1), *MYB15* (c18446_g1), *MYB20* (c16983_g1), *MYB60* (c7787_g2), *MYB52* (c19409_g1), and *MYB4* (c10489_g1)] were selected for qRT-PCR to validate the relative expression level in RNA-seq with Pearson correlation coefficient (*r*). Correlation analysis results (Figure 8) showed the *r*-values of all DEGs ranged from 0.8878 to 0.994, indicating that qRT-PCR expression patterns of the selected DEGs were highly correlated with those in RNA-seq. Thus, our RNA-seq data were reliable. Conclusively, this study proves that the MeJA treatment could trigger extensive transcriptional reprogramming and ultimately enhances CQAs accumulation.

## Discussion

### Regulation of JAs Biosynthesis and Signal Transduction in MeJA-Treated *G. jasminoides* Cells

Jasmonic acid and its derivatives, collectively termed as JAs, are a class of potent lipid-derived phytohormones in plants that mediate responses to many biotic and abiotic stresses and perform various functions of reproduction and metabolic regulation via the signal transduction pathway (Kazan and Manners, 2008). Earlier studies indicated that JAs enhanced the formation of various phenylpropanoids (Prieto and Corchete, 2014; Liu et al., 2018). However, little is known about the specific role of JAs on CQAs accumulation in *G. jasminoides* cells.

Alpha-linolenic acid originating from chloroplast membranes is considered as an important precursor for JAs biosynthesis. Undergoing condensation, cyclization, and β-oxidation reactions, JA were ultimately formed by *LOX*, *AOS*, *AOC*, *OPR*, *ACX*, *ACCA1*, and *MFP2* (Figure 4A; Schaller and Stintzi, 2009). Most of these DEGs showed the upregulated expression in “G0h vs G8h,” “G0h vs G20h,” and “G0h vs G40h.” Specially, *AOC*, which participates in the construction of the characteristic cyclopentanone ring structure of JAs (Jacobo-Velázquez et al., 2015), is pivotal for the JAs biosynthetic pathway and stimulates the accumulation of various secondary metabolites. Overexpression of *AOC* could stimulate endogenous JA accumulation and transcript levels of genes (*PAL*, *C4H*, *4CL*, *COMT*, and *CAD*) related to phenylpropanoid biosynthesis in rice. Meanwhile, higher accumulation of phenolic acids, e.g., cinnamic acid, ferulic acid, and benzoic acid, was also found in the *AOC*-overexpressed transgenic line (Fang et al., 2016). Hence, DEGs in the JAs biosynthetic pathway could modulate endogenous JAs biosynthesis and further trigger the JA signal transduction network.

The bHLH TF *MYC2* is a central regulator involved in the transcriptional regulation of JA-responsive gene expression in the JA signal transduction pathway (Figure 4B), and its expression is repressed by a complex repressor of *JASMONATE ZIM DOMAIN* (*JAZ*) proteins in the absence of a JA signal. Upon sensing of the external stimuli (e.g., the application of exogenous MeJA), the SCF^COI1^-ubiquitin-proteasome pathway is activated and then leads to the degradation of the *JAZ* repressor, which paves the way for *MYC2* to regulate the JA-responsive gene expression (Kazan and Manners, 2008). In our study, we found that six DEGs encoding putative *MYC2* (three DEGs) and *JAZ* (three DEGs) were significantly upregulated by MeJA treatment (Figure 4C). However, DEG of *Col 1* (c20119_g3) showed a downward trend in the three comparisons. It was reported that there were positive and negative feedback regulatory loops to regulate JA signaling. On the one hand, the external stimuli promoted the degradation of the *JAZ* repressor to activate transcription of genes encoding *MYC2*. Moreover, the external stimuli subsequently facilitate the transcription of genes encoding *JAZ*, together with the downregulation of *Col 1*, resulting in resetting the signal transduction pathway and avoiding a runaway response (Kazan and Manners, 2008; Jacobo-Velázquez et al., 2015). In addition, *MYC2* was reported to be a regulator of phenylpropanoid biosynthesis. Salvianolic acid B, a phenolic acid derived from phenylpropanoid, was strongly induced in transgenic *Salvia miltiorrhiza* that overexpressed *SmMYC2*, and expressions of genes for the phenylpropanoid biosynthesis (e.g., *PAL*, *C4H*, and *4CL*) were upregulated in the overexpression lines (Yang et al., 2017).

The complex network of communication among plant hormone signaling pathways is often referred to as hormone cross-talk, which is employed in many plant processes (Shigenaga et al., 2017). Beside the JA signaling pathway, other hormone signaling, such as AUX, CK, BR, GA, ABA, ET, and SA, were also responsive to MeJA treatment in *G. jasminoides* cells (Supplementary Figures 1, 2). Whether hormone cross-talk participates in regulation of CQAs remains to be validated in future studies.

### Candidate Genes Involved in CQAs Biosynthesis and Effects of DEGs on CQAs Accumulation in MeJA-Treated *G. jasminoides* Cells

Chlorogenic acid and its derivatives are derived from the phenylpropanoid biosynthesis pathway in plants, and CQAs in *G. jasminoides* cells consisted of three classes, namely, mono-CQAs (Figures 1C–E), di-CQAs (Figures 1F,G), and its derivatives (Figures 1H,I). It is believed that there might be three distinct pathways for CQAs biosynthesis (Figure 6A). In the initial step, phenylalanine is catalyzed by *PAL* and converted into cinnamic acid. Subsequently, CQAs are synthesized through three putative pathways, which are briefly described as follows: (1) generating CQAs via an activated intermediate (caffeoyl-glycoside); (2) synthesis of CQAs from caffeoyl-CoA and quinic acid by *HQT*; and (3) hydroxylation of *p*-coumaroyl-quinic acid by *C3H* to form CQAs (Villegas and Kojima, 1986; Moglia et al., 2016). The last two pathways for CQA biosynthesis were extensively studied in recent years. Moreover, Lallemand et al. (2012) revealed that the synthesis of di-CQAs could be mediated by *HCT* with mono-CQA and CoA, which was validated *in vitro*. However, the structural genes for di-CQA derivative biosynthesis are still unknown and require further research work (Figure 6A).

In our RNA-seq data, a number of genes encoding *PAL*, *C4H*, *4CL*, *HCT*, and *C3H* were annotated, while none of the sequence in the RNA-seq data was aligned to *HQT*. Thus, the primary biosynthetic pathway for CQAs in *G. jasminoides* cells was speculated as follows: the mono-CQAs biosynthesis might be mainly performed on the basis of pathway (3) mentioned above (involving *PAL*, *C4H*, *4CL*, *HCT*, and *C3H*). Subsequently, di-CQAs were generated by *HCT* from mono-CQA and ultimately converted to di-CQA derivatives in the presence of several unknown structural genes.

A total of 19 DEGs were identified to encode enzymes involved in different steps of mono-CQA biosynthesis, such as *PAL* (nine DEGs), *C4H* (three DEGs), *4CL* (three DEGs), *HCT* (three DEGs), and *C3H* (one DEG), 17 of which showed significantly upregulated expressions over time, and the increased expressions of structural genes were consistent with the mono-CQA accumulation in MeJA-treated *G. jasminoides* cells (Figures 1C–E). Furthermore, the continuously upregulated expression of *C3H* (C18639_g1 and C4961_g1) further converted mono-CQA into di-CQAs (Figures 1F,G). Although the specific structural genes for di-CQA derivatives were still unclear, the enhancement of the precursor supply (mono- and di-CQAs) could be one of the reasons for their increasing accumulation (Figures 1H,I) owing to MeJA elicitation.

### DEGs Involved in Transporter

In plants, the transporter system played a critical role in the process of biosynthesis regulation, transport, and accumulation of the secondary metabolites (de Brito Francisco and Martinoia, 2018). At the cellular level, CQAs were synthesized primarily in chloroplasts and cytoplasm and finally transferred to the vacuole for long-term storage, in which the transport process was involved (Li et al., 2019). The transport of the secondary metabolites in plants mainly comprised membrane transporter-mediated and vesicle-mediated mechanisms (de Brito Francisco and Martinoia, 2018; Li et al., 2019), etc. As for the membrane transporter-mediated mechanism, *ABC* and *MATE* families’ transporter played roles in the sequestration of various phenolic compounds in the vacuole, such as lignin, flavonoid, anthocyanin, and their precursors. An *ABC*-type gene (*MtABCG10*) was responsible for the membrane translocation of *p*-coumaric acid, which was a key precursor for CQAs’ and other phenylpropanoid component’s biosynthesis, and silencing the expression of *MtABCG10* resulted in a lower accumulation of phenylpropanoid-derived medicarpin and its precursors (Biała et al., 2017). Furthermore, the expression of *ABC* transporters could be induced by MeJA elicitation. In *Silybum marianum*, *ABC*-type transporters were proposed to be implicated in the secretion of flavonolignans, and MeJA treatment could stimulate the extracellular flavonolignan accumulation as well as the expression of *ABC*-type transporters (Prieto and Corchete, 2014). *MATE*s, some of which are JAs-responsive secondary metabolite transporters, usually act as transporters for phenolic and other compounds. In *V. vinifera*, a *MATE*-type gene (*VvMATE2*) was assigned as the putative proanthocyanidin transporter expressed during berry development (Pérez-Díaz et al., 2014), and *MATE1* from *Medicago truncatula* was engaged in flavonoid transport (Zhao and Dixon, 2009). Recently, Li et al. (2019) reported a vesicle-mediated mechanism for the CQAs transport in *L. japonica*. Interestingly, some *MATE*-type transporters were required for the vesicle trafficking, and, for instance, the vesicle trafficking of anthocyanidin into vacuole in *V. vinifera* was mediated by two *MATE*-type transporters (*anthoMATEs*) (Gomez et al., 2011). A total of 29 DEGs were identified in the *ABC* and *MATE* superfamilies (Figure 6D) in our RNA-seq data. The differential expression and putative roles of *ABC*s and *MATE*s revealed that they might be associated with response to MeJA and secondary metabolite accumulation, especially for CQAs, in *G. jasminoides* cells. Because information about the specific CQAs transport is still limited, the further functional characterization of the transporters needs to be made out. Thus, our results might provide some useful information concerning transport and accumulation of CQAs in *G. jasminoides* cells.

### Regulation of DEGs Associated With TFs on CQAs Accumulation

Transcription factors, also known as sequence-specific DNA binding proteins, emerge as one of the key factors that modulate the expression of specific genes and the accumulation of messenger RNA at the transcriptional level through sequence-specific DNA binding and protein–protein interactions (Liu et al., 2015). A number of candidate DEGs of TFs were identified in MeJA-treated *G. jasminoides* cells (Figure 7B), some of which (e.g., MYB, bHLH, ERF, and WRKY families) could regulate JAs-induced accumulation of the secondary metabolites (Zhou and Memelink, 2016). TFs of MYB families were proposed to be involved in the positive/negative regulation on biosynthesis of various secondary metabolites, especially on phenylpropanoid biosynthesis (Dubos et al., 2010; Liu et al., 2015). The phenylpropanoid pathway is a complex metabolic network with many shared substrates and branches, and some MYB TFs can simultaneously modulate multiple compounds rather than a single compound, such as chlorogenic acids, flavonoids, and lignins, by regulating the expression of genes in the shared upstream pathway (Tuan et al., 2014).

In terms of the positive regulator from the MYB family (Figure 7C), *MYB85* and *MYB20* TFs from *Arabidopsis thaliana* were developmentally associated with the secondary wall thickening via inducing the expression of *4CL* and *HCT*, which catalyzed the common biosynthetic step of chlorogenic acids and lignin (Figure 6A; Geng et al., 2020). It was reported that *MYB15* could drive lignification through activating the expression of genes related to lignin biosynthesis, e.g., *PAL*, *C4H*, *HCT*, *COMT*, *CCoAOMT*, and *CAD* genes (Chezem et al., 2017). Among them, *PAL*, *C4H*, and *HCT* were the common upstream genes in chlorogenic acids and lignin biosynthesis pathways (Figure 6A). In addition, *MYB103* displayed a positive regulation on the *F5H* expression and S-lignin biosynthesis in *A. thaliana* (Ohman et al., 2012). Moreover, *MYB111*, a R2R3-MYB protein, specially controlled the early steps of the flavonoid biosynthetic pathway catalyzed by *chalcone synthase* (*CHS*), *chalcone isomerase* (*CHI*), *flavanone 3-hydroxylase* (*F3H*), and *flavonol synthase* (*FLS*), and its expression could stimulate flavonoid accumulation (Stracke et al., 2007).

As for the negative regulator from the MYB family, repressing formation of the secondary wall in *MYB52*-overexpressed *A. thaliana* was associated with decreased transcript levels of *4CL* and *CCoAMT* (Karpinska et al., 2004; Qiu et al., 2016). Overexpression of *MYB4* in *A. thaliana* reduced the lignin and flavonoid content due to the reducing expressions of *PAL*, *C4H*, *4CL*, *HCT*, *C3H*, *CCoAMT*, *COMT*, *F5H*, *CCR*, and *CAD*, which included the primary structural gene for CQAs and lignin biosynthesis (Zhu et al., 2013; Figure 6A). Furthermore, *MYB60* repressed the expressions of *flavonoid 3,5-hydroxylase* (*F3′5′H*) and *dihydroflavonol-4-reductase* (*DFR*) in *MYB60*-overexpressed *A. thaliana* and showed a negative regulation on flavonoid accumulation (Park et al., 2008; Oh et al., 2011).

In this work, 36 DEGs were identified and classified to several subgroups of the *MYB* TF family from *A. thaliana*, including four kinds of positive regulators (*MYB85*, *MYB20*, *MYB15*, and *MYB111*) and three kinds of negative regulator (*MYB52*, *MYB4*, and *MYB6*) on phenylpropanoid biosynthesis. Most of the positive regulators displayed a continuously upregulated expression, but most of the negative regulators showed a downtrend in expression after MeJA treatment (Figure 7C), which might be one of the reasons for the upregulation of structural genes for CQAs and lignin biosynthesis (Figure 6). Though the functional characterization of those MYBs and other TFs needs to be verified in the future, the above findings might bring insight into the molecular mechanisms regulating CQAs contents in *G. jasminoides* cells under MeJA elicitation.

## Conclusion

**FIGURE 9:**
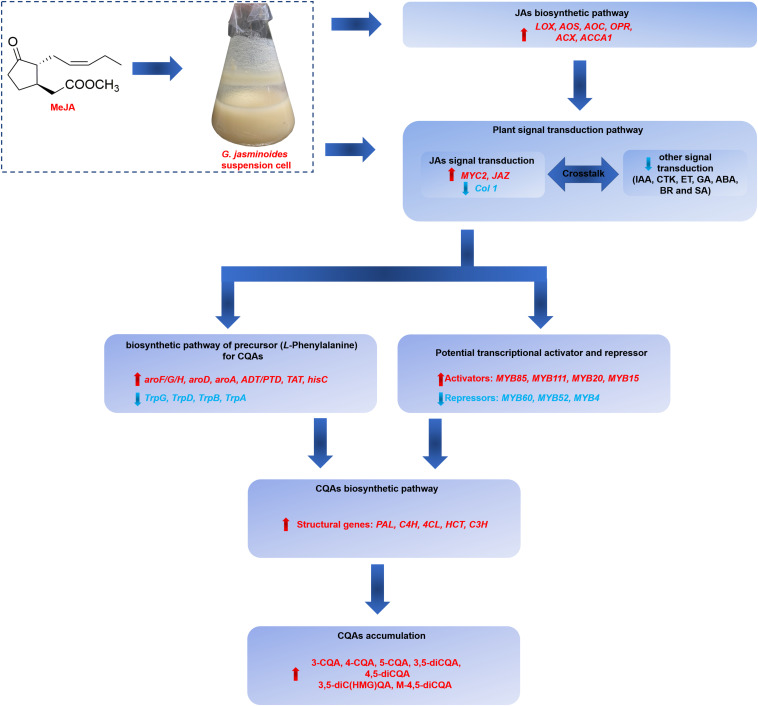
The putative regulatory network of MeJA on CQAs accumulation in *G. jasminoides* cells.

Our work indicated that the application of exogenous MeJA could effectively stimulate the CQAs accumulation in *G. jasminoides* cells. The stimulation mechanism of MeJA was further investigated from the perspective of differential expression of genes, including genes related to JAs biosynthesis, signal transduction, biosynthesis of precursor for CQAs, CQAs biosynthesis, transporters, and TFs. Our RNA-seq analysis of MeJA-mediated transcriptional changes indicated that numerous unigenes in these above-mentioned pathways were differentially expressed, which might be implicated in CQAs biosynthesis and regulation (Figure 9). In MeJA-treated *G. jasminoides* cells, MeJA triggers the expression of genes involved in endogenous JAs biosynthesis (*LOX*, *AOS*, *AOC*, *OPR*, *ACX*, and *ACCA1*) and JAs signal transduction (*MYC2*, *JAZ*, and *Col 1*). Then through the signal transduction network, genes related to biosynthesis of aromatic amino acids, namely, precursors for CQAs, showed an increased expression (*aroF/G/H*, *aroD*, *aroA*, *ADT/PTD*, *TAT*, and *hisC*), which might boost supply of the precursors. Meanwhile, TFs, especially for the MYB family, showed significant response to MeJA treatment and might display positive (*MYB85*, *MYB20*, *MYB15*, and *MYB111*) or negative (*MYB52*, *MYB4*, and *MYB6*) regulations on CQAs biosynthesis, resulting in increased expression of key genes for CQAs biosynthesis (*PAL*, *C4H*, *4CL*, *HCT*, and *C3H*). Ultimately, the accumulation of CQAs in *G. jasminoides* cells was significantly increased. Moreover, our data will surely provide a massive genetic resource for further investigation of CQAs biosynthesis and lay the foundations for genetic engineering to boost the yield of these important compounds in *G. jasminoides* cells.

## Data Availability Statement

The data presented in the study are deposited in the (NCBI Sequence Read Archive) repository, accession number (PRJNA672865).

## Author Contributions

ZL: methodology, validation, investigation, data curation, visualization, writing—original draft, and writing—review and editing. AM: formal analysis and writing—review and editing. ZW: methodology, validation, and writing—review and editing. XZ: methodology and validation. YZ and LC: supervision and validation. MG: supervision, project administration, and writing—review and editing. ZY: supervision, funding acquisition, project administration, and writing—review and editing. All authors contributed to the article and approved the submitted version.

## Conflict of Interest

The authors declare that the research was conducted in the absence of any commercial or financial relationships that could be construed as a potential conflict of interest.

## Footnotes

1 http://plntfdb.bio.unipotsdam.de/v3.0/

## Abbreviations

*G. jasminoides*: *Gardenia jasminoides* Ellis
CQAs: chlorogenic acid and its derivatives
JAs: jasmonate
JA: jasmonic acid
MeJA: methyl jasmonate
RNA-seq: RNA sequencing
DEGs: differentially expressed genes
TFs: transcription factors
PAL: phenylalanine ammonia lyase
C4H: cinnamate 4-hydroxylase
4CL: 4-coumarate CoA ligase
HCT: hydroxycinnamoyl CoA shikimate/quinate hydroxycinnamoyl transferase
C3H: *p*-coumarate 3 ′ -hydroxylase
UGCT: UDP glucose: cinnamate glucosyl transferase
HCGQT: hydroxycinnamoyl D-glucose: quinate hydroxycinnamoyl transferase
COMT: 3-*O*-methyltransferase
CCoAMT: caffeoyl-CoA *O*-methyltransferase
F5H: ferulate-5-hydroxylase
CCR: cinnamoyl-CoA reductase
CAD: cinnamyl-alcohol dehydrogenase
LOX: lipoxygenase
AOS: hydroperoxide dehydratase
AOC: allene oxide cyclase
OPR: 12-oxophytodienoic acid reductase
ACAA1: acetyl-CoA acyltransferase 1
JAZ: JASMONATE ZIM DOMAIN
MYC2: bHLH transcription factor MYC2.
