# Supplementary material for: Enhanced Biosynthesis of Chlorogenic Acid and Its Derivatives in Methyl-Jasmonate-Treated Gardenia jasminoides Cells: A Study on Metabolic and Transcriptional Responses of Cells
Source: Front Bioeng Biotechnol. 2021 Jan 5;8:604957. doi: 10.3389/fbioe.2020.604957 (PMC7813945; doi:10.3389/fbioe.2020.604957)
Supplement: Supplementary file 1 [file Data_Sheet_1.doc]

**Table S1** Primers for qRT-PCR

| Primer name | Primer sequence |
| --- | --- |
| UBQ5-F | TGGGAGAACCCTTGCTGATT |
| UBQ5-R | AGCGTTATGGTCTTCCCTGTC |
| AOS-F | CTCATATTCGCCACCTGCTT |
| AOS-R | TCGCTCTCATTGTGACTCTGC |
| JAZ-F | GAAATCAAACTTTGCCCACACT |
| JAZ-R | GCTGACCCGAAATCTCCAT |
| MYC2-F | TCCAGACCCAGTCAGATTGCT |
| MYC2-R | TTGGTCCCTTGGAAGTAGCC |
| PAL-F | TTTGGACTATGGTTTCAAGGGT |
| PAL-R | CCACGGCTTCTGCTGTTTT |
| C4H-F | AGTCACTGAACCAGACACCCAT |
| C4H-R | TCTCGGCTGAAATATCATACCC |
| 4CL-F | CCAGTTCAGCAACCGTCCTT |
| 4CL-R | GCTTGTAGAATGGATTGGCAGT |
| HCT-F | CAAGGGATGAAGAAGGAAGGAT |
| HCT-R | TTGAAACGAGTCACCTGGAAA |
| C3H-F | GGGTCAGTAGCATTCAACAACA |
| C3H-R | AAACATCCAACGAAGCCAAG |
| MYB85-F | GGTTACAACGTACCCAGAGACAC |
| MYB85-R | GAACGCCAGCACAAATCCT |
| MYB111-F | GCCCAGTCATCTTCACAATA |
| MYB111-R | ATCACTCACGGGAGCCAC |
| MYB15-F | CCAGGACGAACCGACAAC |
| MYB15-R | GGCATCCGTGAAGGATAAG |
| MYB20-F | ATTGCTTCTCATCTCCCTG |
| MYB20-R | CTTCTCCTTTCGCCTCTG |
| MYB60-F | CTCAAATGCTGGATAGGAA |
| MYB60-R | TTAGGAGGCAATGGGACA |
| MYB52-F | TCTTTCCATCACTGCCTTTA |
| MYB52-R | GCACCCAACACTCCATACA |
| MYB4-F | CGGTGAAGCTACAAAGAAAT |
| MYB4-R | TGATAAGCCTCCCTAACTAAA |

**Table S2** Results of RNA-Seq evaluation

| Sample_name | G8h | G20h | G0h | G40h |
| --- | --- | --- | --- | --- |
| Total reads | 5,741,626 | 8,263,184 | 7,394,830 | 21,010,198 |
| Total mapped | 3,855,586 (67.15%) | 5,948,742 (71.99%) | 5,583,824 (75.51%) | 17,037,760 (81.09%) |
| Mutiple mapped | 933,628 (16.26%) | 1,198,724 (14.51%) | 1,201,908 (16.25%) | 2,612,462 (12.43%) |
| Unique mapped | 2,921,958 (50.89%) | 4,750,018 (57.48%) | 4,381,916 (59.26%) | 14,425,298 (68.66%) |
| Read1 mapped | 1,460,979 (25.45%) | 2,375,009 (28.74%) | 2,190,958 (29.63%) | 7,212,649 (34.33%) |
| Read2 mapped | 1,460,979 (25.45%) | 2,375,009 (28.74%) | 2,190,958 (29.63%) | 7,212,649 (34.33%) |
| Mapped to '+' | 14,60,979 (25.45%) | 2,375,009 (28.74%) | 2,190,958 (29.63%) | 7,212,649 (34.33%) |
| Mapped tp '-' | 1,460,979 (25.45%) | 2,375,009 (28.74%) | 2,190,958 (29.63%) | 7,212,649 (34.33%) |
| Non-splice reads | 2,921,958 (50.89%) | 4,750,018 (57.48%) | 4,381,916 (59.26%) | 14,425,298 (68.66%) |
| Splice reads | 0 (0.00%) | 0 (0.00%) | 0 (0.00%) | 0 (0.00%) |
| Reads mapped in proper pairs | 2,921,958 (50.89%) | 4,750,018 (57.48%) | 4,381,916 (59.26%) | 14,425,298 (68.66%) |

Total Reads: the number of all clean data form each library

Total mapped: the number of reads that can be mapped to the reference sequences

Mutiple mapped: the number of reads with multiple alignment positions on the reference sequences

Unique mapped: the number of reads with unique alignment position on the reference sequences

Read1/ Read1 mapped: the number of reads form read1/read2 mapped to the reference sequences (only calculate unique mapped sequences)

Mapped to '+'/Mapped tp '-': the number of reads that can be mapped to positive/negative chains of reference sequences (only calculate unique mapped sequences)

Non-splice reads: the number of reads whose whole sequence are aligned to the exons

Splice reads: the number of reads that can be segmentally aligned to the reference sequences

Reads mapped in proper pairs: the number of paired reads that can be simultaneously aligned to the reference sequences

Note: we ran the RSEM program and invoked Bowtie with parameters of -v 2 -S for RNA-Seq evaluation. All the spliced unigenes after assembly from each library were regard as reference sequences. Clean reads from each library were aligned to reference sequences and then statistical results were obtained

**Figure S1**


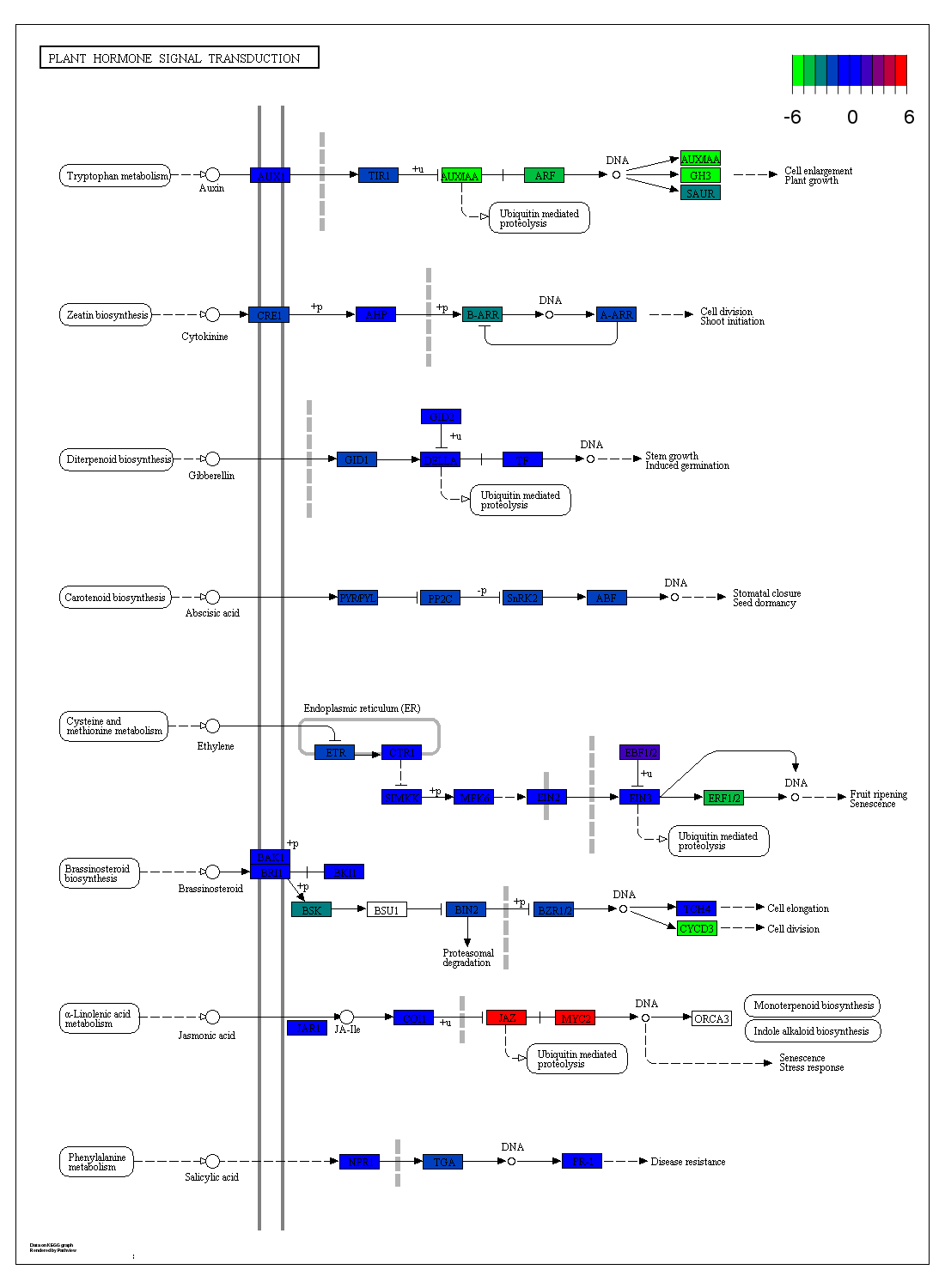


Note: all DEGs in plant hormone signal transduction pathway were mapped to KEGG pathway of ko04075, and different colors of rectangles meant relative expression of DEGs.

**Figure S1** Plant hormone signal transduction pathway

**Figure S2**


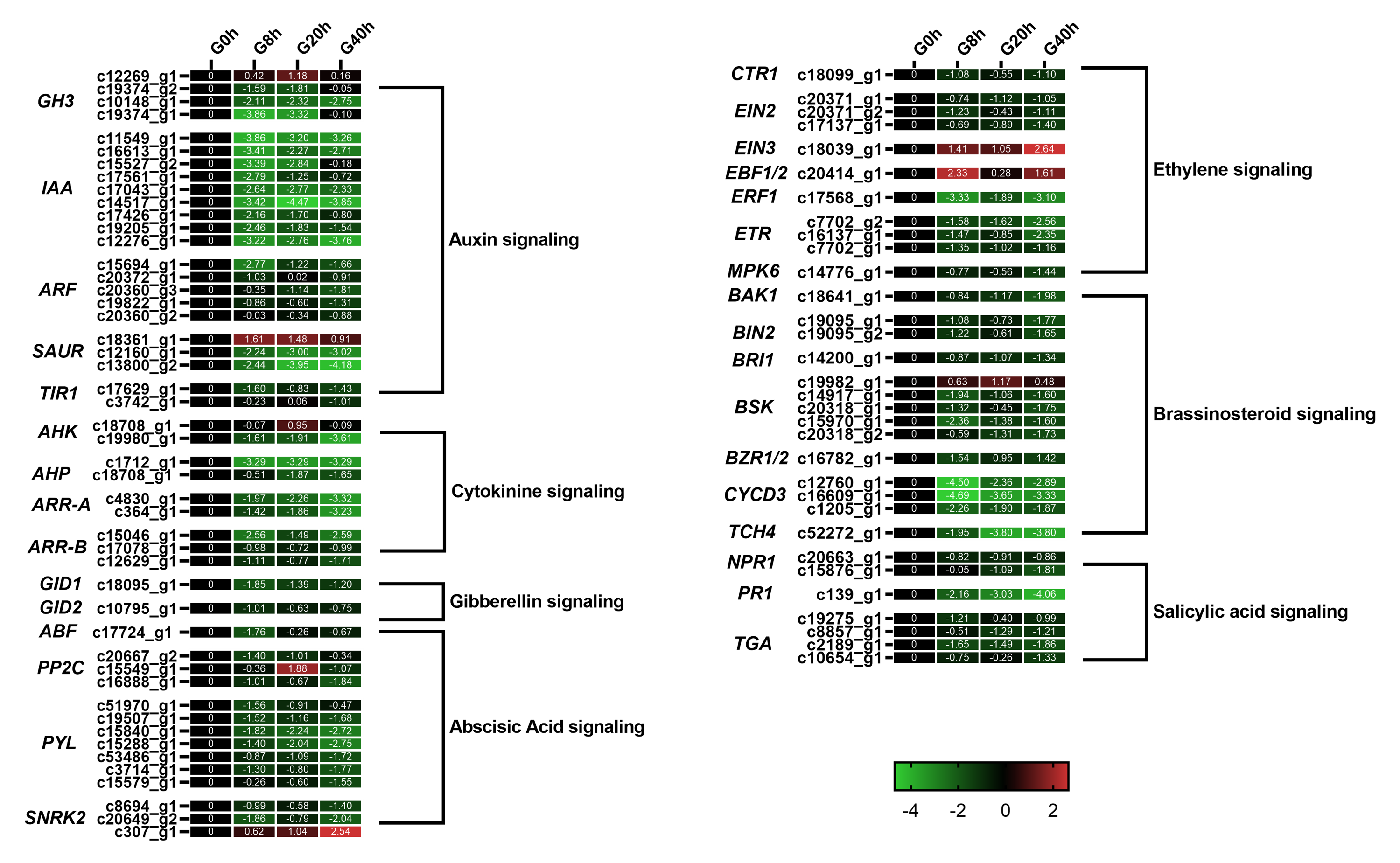


**Figure S2** Expression heatmap of DEGs in plant hormone signal transduction pathway
